# Two levels above and one level below pedicle screw fixation for the treatment of unstable thoracolumbar fracture with partial or intact neurology

**DOI:** 10.1186/1749-799X-4-28

**Published:** 2009-07-27

**Authors:** Hitesh N Modi, Kook Jin Chung, Il Woo Seo, Hoi Soo Yoon, Ji Hyo Hwang, Hong Kyun Kim, Kyu Cheol Noh, Jung Han Yoo

**Affiliations:** 1 Department of Orthopedics, Kangnam Sacred Heart Hospital, College of Medicine, Hallym University, Seoul, Korea; 2 Department of Radiology, Hallym Sacred Heart University Hospital, College of Medicine, Hallym University, Seoul, Korea

## Abstract

**Background:**

Treatment of unstable thoracolumbar fractures is controversial regarding short or long segment pedicle screw fixation. Although long level fixation is better, it can decrease one motion segment distally, thus increasing load to lower discs.

**Methods:**

We retrospectively analyzed 31 unstable thoracolumbar fractures with partial or intact neurology. All patients were operated with posterior approach using pedicle screws fixed two levels above and one level below the fracture vertebra. No laminectomy, discectomy or decompression procedure was done. Posterior fusion was achieved in all. Post operative and at final follow-up radiological evaluation was done by measuring the correction and maintenance of kyphotic angle at thoracolumbar junction. Complications were also reported including implant failure.

**Results:**

Average follow-up was 34 months. All patients had full recovery at final follow-up. Average kyphosis was improved from 26.7° to 4.1° postoperatively and to 6.3° at final follow-up. And mean pain scale was improved from 7.5 to 3.9 postoperatively and to 1.6 at final follow-up, All patients resumed their activity within six months. Only 4 (12%) complications were noted including only one hardware failure.

**Conclusion:**

Two levels above and one level below pedicle screw fixation in unstable thoracolumbar burst fracture is useful to prevent progressive kyphosis and preserves one motion segment distally.

## Background

The thoracolumbar junction is the most common site of spinal injuries. The surgical treatment of unstable fractures and fracture dislocations of thoracolumbar spine remains controversial [1]. The goals of treatment in thoracolumbar fractures are restoring vertebral column stability and obtaining spinal canal decompression, leading to early mobilization of the patient. Either anterior, posterior or both approaches can be used to achieve fusion but the efficacy of either approach is the same [2,3]. However, posterior approach is less extensive, and most spine surgeons advocate posterior fusion as the treatment of choice for unstable thoracolumbar injuries [4,5]. The importance of early decompression and stabilization of unstable vertebral injuries has been emphasized in several reports [4,6]. Pedicle screw devices allow immediate stable fixation as the screws traverse all the three columns. Short-segment (SS) pedicle instrumentation has become a popular method since Dick et al [7] introduced the "fixateur interne" device. Various techniques have since arisen for the management of unstable thoracolumbar fractures. Nowadays, controversy still exists over whether SS pedicle instrumentation is a suitable method. Biomechanical and clinical studies, however, have shown that when there is loss of more than 50% of the vertebral body height or angulations of the thoracolumbar junction of more than 20° [8], acute spinal instability results, and the spinal segment will eventually fail with weight-bearing. Biomechanical studies have also shown that spinal instability results when there is a failure of at least two of Denis three columns [9]. Gurr et al [10] found that CD instrumentation placed two levels above and two levels below the injured level in an unstable calf spine model provided more stiffness than that in the intact spine. Krag [11] has suggested segmental pedicle fixation two levels above the kyphosis to prevent implant failure. Carl et al [4] reported that segmental transpedicular fixation two levels above the kyphosis should be used at the thoracolumbar junction where compressive forces act more anteriorly, whereas in the more lordotic middle and lower lumbar spine where the compressive forces act more posteriorly, no implant failure occurred with use of the one above-one below construct.

Here we present our results in unstable thoracolumbar fracture patients who were treated with pedicle screw fixation two level above and one level below the fracture vertebra, in order to preserve motion segment below the level of fracture. The purpose was to study the effectiveness of pedicle screw fixation, two levels above and one level below the fractured vertebra, in order to prevent postoperative kyphosis and high implant failure rate.

## Methods

We retrospectively reviewed the results of unstable thoracolumbar fractures with partial or intact neurology in consecutive 31 patients who were operated between June 2004 and June 2006 at our institute by a single spine surgeon (Table 1). There were 18 males and 13 females with an average age of 40.6 ± 12.7 (range, 19~63 years). There were 7, 13 and 11 patients who had fractures at T11, T12 and L1 level, respectively. 22 patients had injury due motor vehicle accident (MVA) and 9 had injury due to fall from height. Neurologic compromise was graded according to Frankel. There were 4, 7 and 20 patients with Frankel grade C, D and E respectively. A postoperative neurologic examination was also performed in all patients 1 year later and the findings compared with the preoperative examination. Following a routine examination and X-ray of the spine, computed tomography (CT) scan of the involved vertebra and adjacent vertebrae was carried out. McAfee's [12] system was used to classify the fractures. There were 14 unstable burst (UB) fractures, 9 translational (TRS) injuries and 8 flexion-distraction (FD) injuries. Frankel's grade system was used for assessment of neurological deficit on admission and subsequently in the follow-up.

**Table 1:** Demographics of each patient with preoperative and postoperative kyphotic angle, neurological status and pain scale.

**No**	**Sex**	**F-U (months)**	**Age (years)**	**Level**	**Injury (cause)**	**McAfee (type)**	**ISI (Days)**	**Kyphosis angle**			**Neurology (Frankel)**		**Pain Scale (Dennis)**		
								
								**Preop**	**IMPO**	**Final**	**Preop**	**Final**	**Preop**	**IMPO**	**Final**
1	M	44	19	L1	MVA	FD	2	24	0	2	E	E	7	3	1
2	M	43	44	T11	MVA	UB	0	30	2.9	5.8	E	E	8	4	2
3	F	42	28	T12	MVA	UB	0	24.1	2	5.2	C	E	7	4	1
4	M	42	60	L1	MVA	UB	1	25.3	2	4.5	E	E	9	5	2
5	F	36	43	T12	MVA	UB	6	27.4	2.4	5.1	D	E	7	4	1
6	F	30	54	T12	MVA	UB	1	28.9	4.3	6.4	E	E	9	5	2
7	M	28	42	T12	MVA	UB	1	25.2	3	5.8	E	E	8	5	2
8	F	27	24	T11	MVA	UB	3	30.1	4.5	7.1	D	E	9	4	3
9	M	49	28	T12	MVA	UB	1	29.5	3.4	5.7	E	E	7	4	1
10	M	24	39	T12	Fall	TRS	1	29.3	4.2	5.8	D	E	7	5	1
11	M	48	63	L1	Fall	UB	1	29.3	5.3	7.7	E	E	8	4	2
12	F	47	50	L1	MVA	FD	5	29	5	6.5	C	E	8	5	2
13	M	44	39	T12	Fall	TRS	1	31.5	3.5	6.1	E	E	9	6	2
14	M	29	42	L1	MVA	UB	0	25.4	4.2	6.9	D	E	6	5	2
15	M	28	39	T12	MVA	UB	0	27.5	3.3	5.6	E	E	7	3	1
16	M	26	40	L1	Fall	TRS	10	26	10.5	16.4	E	E	6	3	1
17	F	26	59	T12	MVA	TRS	7	20.6	5.4	8.1	C	E	6	4	2
18	F	46	33	T11	MVA	TRS	4	20.5	7.3	8.6	E	E	7	2	1
19	F	42	26	T12	Fall	TRS	6	24	6	7.4	E	E	7	2	1
20	M	24	24	T11	MVA	FD	3	23	5	6.8	E	E	7	3	2
21	M	34	48	L1	MVA	FD	1	20.3	3.5	5.9	D	E	6	3	1
22	M	33	30	T12	Fall	FD	5	32.3	5.7	7.6	E	E	9	4	3
23	F	33	55	T12	MVA	TRS	4	31.5	2.5	3.8	E	E	8	3	1
24	M	32	62	L1	MVA	FD	1	24	4.3	5.8	E	E	7	4	1
25	F	32	23	L1	Fall	FD	8	21.8	4	5.8	D	E	7	3	2
26	F	36	43	T11	MVA	UB	6	22.8	3	5.2	E	E	7	3	2
27	M	24	23	T11	Fall	UB	3	20.5	4.6	6.5	E	E	7	3	1
28	M	24	56	T11	Fall	TRS	4	24.3	0	3.4	E	E	7	4	1
29	F	27	42	L1	MVA	TRS	7	35	8.4	9.8	D	E	9	4	2
30	F	26	37	L1	MVA	UB	3	30.3	3.7	4.6	E	E	9	5	2
31	M	29	42	T12	MVA	FD	14	35	3.2	5.9	C	E	8	5	3

Abbreviations: F-U: Follow-Up; ISI: Injury Surgery Interval; IMPO: immediate post operative; MVA: motor vehicle accident; UB: Unstable burst fracture; TRS: translational injury; FD: flexion-distraction injury; HWF: hard ware failure; SWI: superficial wound infection.

**Figure 1:**
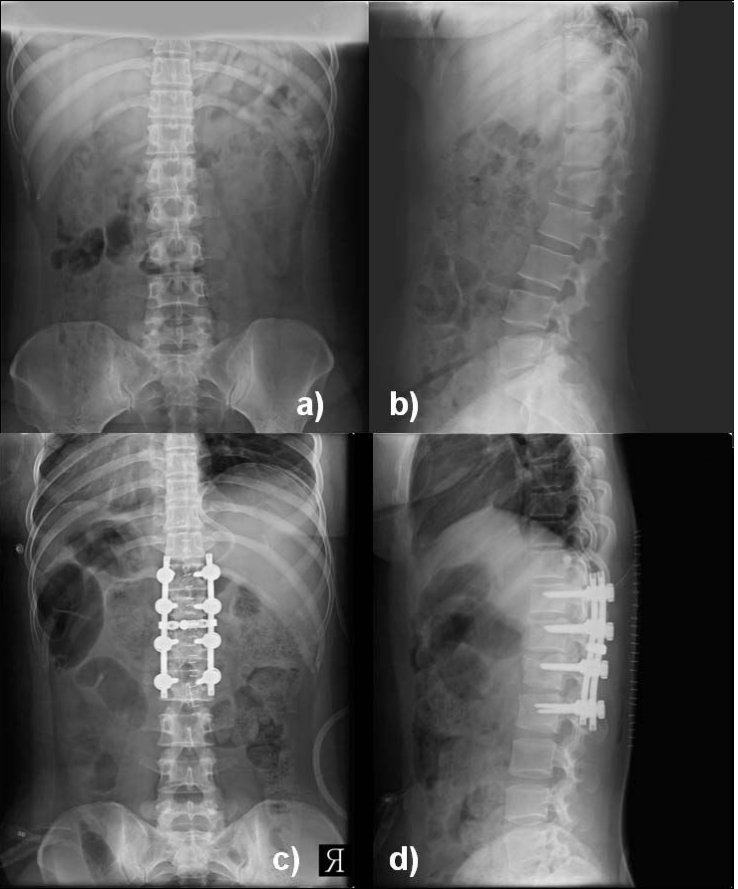
**showing preoperative a) AP and b) lateral radiogram of a patient with unstable L1 fracture with kyphosis of 28-degrees**. Immediate postoperative c) AP and d) lateral radiogram showed correction of kyphosis.

**Figure 2:**
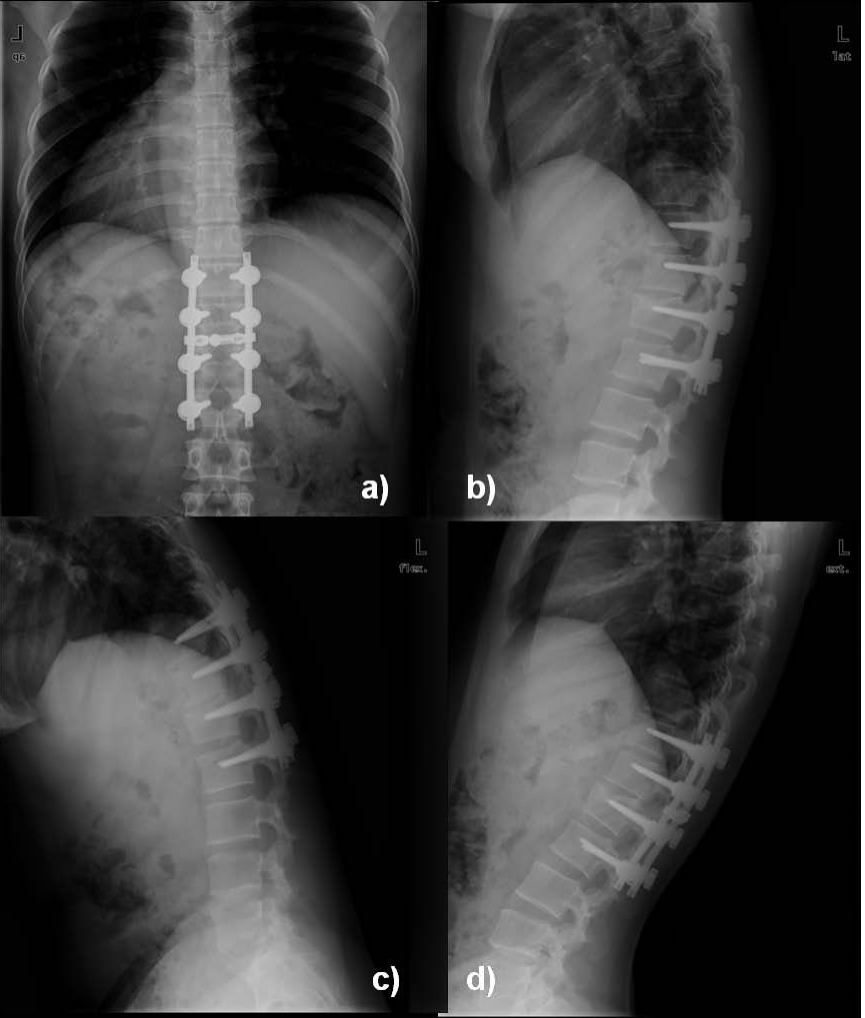
**at final follow-up (3 years) a) AP and b) lateral radiogram showed maintenance of correction in kyphosis, and c) flexion and d) extension view showed good lumbar motion with maintenance of correction**.

Indication for surgical stabilization in patients who had partial or intact neurology was based on instability criteria of kyphotic deformity of more than 20° and/or vertebral body height loss of more than 50 compared to vertebra below. The senior author (KJC) performed all surgeries. At surgery, the patients were placed in a hyperextended prone position with the abdomen hanging free, thus preventing excessive intraoperative bleeding and achieving a significant initial reduction of the spinal fracture. All patients were operated with single stage posterior surgery using pedicle screw instrumentation, two levels above and one level below the fractured vertebra (Figs. 1 and 2). All pedicle screws were inserted under C-arm guidance. The rod was then fixed two levels above the fracture into the four screws and after that, torque was applied through the rod pusher to bring the vertebra back to the rod. Gentle distraction at the level of the fracture followed, restoring tension to the posterior longitudinal ligament (ligamentotaxis) and thereby, achieving anatomic reduction. None of our patients underwent discectomy and/or laminectomy and decompression procedure. All the patients had cross link fixation across the fracture site for preventing windshiled effect of construct with at least one pedicle screw fixation in the fractured vertebra. After the fixation, posterior fusion achieved using cancellous bone grafts harvested from posterior iliac crest.

All patients were managed with immobilization in a custom-molded thoracolumbosacral brace for three months postoperatively. All patients with age more than 40 years were investigated in form of DEXA (Dual Emission X-ray Absorptiometry) to evaluate BMD (bone mineral density) as a routine protocol in our clinic for the treatment of osteoporosis. The patients were followed with serial physical examinations and radiographs. With the use of lateral radiographs centered over the fracture level, kyphosis or lordosis was measured from the superior end-plate of the intact vertebra cephalad to the fracture to the inferior end-plate of the vertebra caudad to the fracture. Progression was considered to be absent, minor, or major. Minor progression was defined as kyphosis measuring 5 to 10° more at the time of follow-up than it had on the immediate postoperative radiographs. Major progression was defined as an increase of 10° or more compared with the measurement on the initial postoperative radiographs. Denis pain score were also compared statistically for each patient, immediate postoperatively and at the final follow-up, with preoperative pain score.

## Results

Average follow-up was 34 ± 8.2 months (range, 24~49 months). Mean injury surgical interval was 3.5 days ranging from minimum 6 hours to maximum 14 days. Table 1 presents a master chart on the patients and their treatment, including patient parameters, mode of trauma, level of injury, type of fracture, injury surgery interval, kyphotic angles (pre-operative, post-operative, loss of kyphosis), neurological status, complications and pain evaluations.

The average pre-operative kyphotic angle was 26.7° which improved to 4.1° in the immediate post-operative period suggesting 84.6% correction (p < 0.0001, paired t-test) (Fig 1). At final follow-up it was 6.3° suggesting 76.4% correction (p < 0.0001, paired t-test) (Fig 2). Considering the change according to degree of progression, all but one patients showed no progression of kyphosis more than 5-degree at final follow-up. Only one patient (patient 18) showed minor progression (6.1°) at the final follow-up. The average pre-operative vertebral height was 41.9% compared to the vertebra below the fracture, which improved to 76.2% in the immediate postoperative period. The loss of body height averaged 2.7% at the final follow-up, and the loss of kyphotic correction averaged 2.2°. Similarly, the average preoperative pain scale (Denis) was 7.5, which was improved to 3.9 (49.1%) immediate postoperatively. At final follow-up mean pain scale was 1.6 suggesting overall improvement of 79.1% than preoperative level. Our findings also suggested continuous improvement in pain scale after the surgery. All the patients had Frankel grade E neurology at final follow-up suggesting all the patients were improved in neurological status after the surgery.

There were only four complications (12%). Two patients (patient 12 and 19) had screw loosening. One was 63-years-old male and other was 59-years-old female. They were osteoporotic patients which were confirmed with bone densitometry. Therefore screw loosening in them was thought to be due to osteoporosis. None of them had any deterioration in their functional activity. One 40 years male (patient 18) had breakage of right L1 screw; however there was no movement noted on flexion-extension radiogram and patient was symptomless. No treatment was done for the hardware failure in that patient. Finally, one 42-years-old male (patient 35) developed superficial wound infection which was treated with repeated dressings and parenteral antibiotics. His further follow-up was uneventful. All patients were able to return to their previous activity within six months of surgery and none of the patient had deterioration in neurology on regular follow-up. At final follow-up there was no instability detected on the flexion-extesion radiogram in all patients.

## Discussion

Posterior transpedicular screw fixation initially was reported by Boucher in 1959 [13]. Since then, modern instrumentation systems have been developed. These systems control segmental motions in three dimensions, preserve motion segments, avoid long fusions, and provide a more stable construct [5]. As with all surgical implants; transpedicular screw instrumentation maintains reduction until bony union is achieved. Short-segment posterior fixation (SSPF) is the most common and simple treatment, offering the advantage of incorporating fewer motion segments in the fusion [14-17]. A review of the literature showed that SSPF alone led to a 9–54% incidence of implant failure and re-kyphosis in the long-term follow-up, and 50% of the patients with implant failure had moderate-to-severe pain [18,15,19]. To prevent this, several techniques have been developed to augment the anterior column in burst fractures, such as transpedicular bone grafting [18,20,15], placement of body augmenter [16], polymethylmethacrylate (PMMA) injection [21], anterior instrumentation and strut grafting [3], or long-segment posterior fixation (LSPF) [14,22]. In current study we retrospectively analyzed all patients who were treated with posterior approach using pedicle screw fixation two levels above and one level below the fracture vertebra, to study the effectiveness of fixation in preventing postoperative development of kyphosis and hardware failure.

Although SS pedicle instrumentation has been considered as a superior method, [7,23] several studies showed that SS pedicle instrumentation had a high rate of failure [18,19]. Nevertheless, some studies demonstrated that clinical long-term results were favorable in patients who underwent SS pedicle instrumentation [23]. McLain et al [19], in their report of early failure of SSPF for thoracolumbar burst fracture noted three kinds of hardware failure with this fixation: progressive kyphosis secondary to the bending of screws, kyphosis secondary to osseous collapse or vertebral translation without bending of the hardware, and segmental kyphosis after a caudad screw in the lumbar construct broke. And they noted that untreated anterior instability, and pre-stressing of the screws when the rods were contoured in situ, resulted in a high rate of failure. Altay et al [24] reported that use of four pairs of screws (two above and two below) to lengthen the level arm of the construct would probably not only enhance the stability but also allow effective reduction of kyphotic deformity. SSPF alone can give good clinical and radiological outcomes for certain fractures in the thoracolumbar junction. Detection of such fractures in which SSPF, without supporting anterior column, is sufficient and does not lead to implant failure and correction loss. Tezeren and Kuru [25], in their study comparing short segment versus long segment fixation in thoracolumbar burst fractures, demonstrated that LS instrumentation is an effective way to manage thoracolumbar burst fractures. SS pedicle instrumentation had a high rate of failure. However, LS instrumentation prolonged the operative time and increased the amount of blood loss significantly. De Peretti et al [20] suggested that fixation by screw and hook constructs, gripping the two vertebrae above the lesion and screws and hooks gripping the first vertebra below the lesion, was an effective way to stabilize thoracolumbar junction burst fractures. Carl et al [4] also reported that segmental transpedicular fixation two levels above the kyphosis should be used at the thoracolumbar junction where compressive forces act more anteriorly. Therefore we prefer to put the pedicle screw two levels above the fracture site in order to prevent progressive kyphosis as well as hard ware failure. On the other hand preferring one level fixation distal to fracture site was to preserve the motion segment as much as possible in the lumbar level. Our result showed that we have achieved both our purposes with this fixation.

As all of the patients in our study had partial or intact neurology at the time of presentation, decision to operate was taken if they had kyphosis angle more than 20-degree and/or anterior vertebral body height more than 50%; which suggested unstable fractures. Average preoperative kyphosis angle was 26.7° preoperatively which improved to 4.1° immediate postoperatively and maintained at 6.3° at final follow-up. Our results suggested a success similar to LSPF construct reported in the literature. And we agree that fixing the fracture two levels above prevents progressive kyphosis development. Additionally evaluation of pain scale for all the patients suggested that our fixation strategy had been successful in improving the pain scales at final follow-up and all the patients were able to go back to their previous level of activity after the operation.

Butt et al [26] recently reported success of short segment pedicle screw fixation in thoracolumbar burst fractures; however the 40% (20 out of 50 patients) hardware failure rate that they reported is worrisome. We think that this high rate of implant failure is probably due to SSPF. In the present study we had only 12% (four patients out of 31) complication rate; one superficial wound infection, two screw loosening and one screw breakage. Screw loosening was found in patients with elder age which was probably due to their poor bone quality and therefore only one patient (42-years-old male) developed implant failure which is quite low than SSPF in the literature. We think that two level above and one level below pedicle screw fixation has high success rate in preventing hardware failure related complication similar to LSPF; and additionally we could save one motion segment distally as well in all the patients.

As our patients had partial neurological injury or intact neurology, we could preserve the lamina and other posterior structures in all patients. We feel that further study comparing the effectiveness of this construct in patients with laminectomy would be helpful to confirm the success of this technique. We think this could be the only weak factor in our study and we recommend further study in laminectomized patients in future. Additionally, we could not evaluate the union in all patients because all subjects did not undergo for CT scan at final follow-up. However, there was no instability noted on flexion-extension radiogram at final follow-up.

## Conclusion

Our study reported prevention of progressive kyphosis development with the use of two levels above and one level below pedicel screw construct in unstable thoracolumbar burst fractures in patients with partial or intact neurology. Additionally it is helpful to preserve one motion segment distally at lumbar level and improving the pain scale at final follow-up.

## Competing interests

The authors declare that they have no competing interests. Each author certifies that he has no commercial associations (e.g. consultancies, stock ownership, equity interests, patent/licensing arrangements, etc) that might pose a conflict of interest in connection with the submitted article.

## Authors' contributions

HNM has contributed in conception and design of data, analysis and interpretation of data, drafting the manuscript and revising it critically, KJC has contributed in conception and design of data, drafting the manuscript and given the final approval of manuscript, IWS has contributed in drafting the manuscript and data analysis, HSY has contributed acquisition of data and revising it critically, JHH has contributed in acquisition of data, revising the manuscript critically and given the final approval, HKK has contributed in drafting the manuscript and designing of data and revising it critically, KCN has contributed in acquisition of data and analysis and interpretation of data, and JHY has contributed in acquisition and analysis of data. All authors read and approved the final manuscript.
